# Global Temporal Trends and Projections of Acute Hepatitis E Epidemiology for Adults 65 Years and Older from 1990 to 2021: Global Burden of Disease 2021 Based Study

**DOI:** 10.3390/tropicalmed11030082

**Published:** 2026-03-17

**Authors:** Shuangshuang Ma, Qingling Wang, Junjie Lin, Yufeng Gao

**Affiliations:** 1 Department of Infectious Diseases, The First Affiliated Hospital of Anhui Medical University, No. 218 Jixi Road, Hefei 230031, China; 18756551698@163.com (S.M.); linjunjieaust@163.com (J.L.); 2 Anhui Province Key Laboratory of Infectious Diseases, Anhui Medical University, No. 218 Jixi Road, Hefei 230031, China; 3 Department of Geriatric Respiratory Medicine, The First Affiliated Hospital of Anhui Medical University, No. 218 Jixi Road, Hefei 230031, China; w18270343814@163.com

**Keywords:** acute hepatitis E, population aging, GBD, Bayesian projections, age-period-cohort analysis, Bayesian age-period-cohort

## Abstract

Background: Acute hepatitis E (AHE) poses escalating risks to older adults (≥65 years), compounded by immunosenescence and comorbidities. Using Global Burden of Disease (GBD) 2021 data, this study analyzes global AHE burden, trends, and projections in aging populations. Methods: Age-standardized rates (ASIR, ASMR, ASDR) for AHE in adults ≥ 65 years were extracted from GBD 2021 across 204 countries (1990–2021). Frontier analysis assessed gaps between observed burdens and sociodemographic index (SDI)-based theoretical minima. Age-period-cohort (APC) modeling evaluated age/period/cohort effects. Bayesian (BAPC), NORDPRED, and ARIMA models projected trends to 2050. Results: Global ASIR increased by 1.5% annually (1990–2021), with ASMR and DALYs declining significantly. Middle SDI regions showed the steepest ASIR rise (net drift: 0.064%/year), while high SDI areas had volatile trends. Age effects peaked in ≥95-year-olds. Frontier analysis revealed persistent ASIR-SDI gaps, particularly in low-middle SDI regions. Projections indicate a ASIR rise by 2050 (113.04/100,000), contrasting with declining ASMR (0.056/100,000) and ASDR (1.31/100,000) and the NORDPRED, ARIMA, and EAPC models exhibit analogous global predictive trends. Conclusions: Diverging trends of rising incidence and falling mortality highlight unmet prevention needs. High-burden regions require SDI-stratified strategies, prioritizing vaccination programs (e.g., HEV 239), zoonotic transmission control, and enhanced surveillance. The Sustainable Development Goals (SDGs) envision hepatitis elimination by 2030 (Target 3.3). However, our analysis projects ongoing AHE burden in aging populations through 2050, indicating the need for post-2030 policy adaptations.

## 1. Introduction

Hepatitis E virus (HEV) infection, historically characterized as a self-limiting hepatotropic disease predominantly affecting younger demographics, has emerged as a pressing public health threat amid the global aging transition. By 2050, adults aged ≥ 65 years are projected to account for nearly 20% of the global population, and this cohort exhibits disproportionately severe clinical outcomes following HEV infection, with mortality rates substantially higher than those observed in younger age groups. This heightened vulnerability is primarily attributable to age-related immunosenescence and the high prevalence of comorbid conditions, most notably chronic liver disease [1,2,3,4]. This demographic shift underscores the alignment with the World Health Organization (WHO)’s “Decade of Healthy Aging (2021–2030)” initiative, which prioritizes the mitigation of infectious disease burdens among elderly populations as a core public health objective [5]. However, the epidemiological profile of acute hepatitis E (AHE) in older adults remains incompletely elucidated, often confounded by region-specific studies with limited generalizability and methodological constraints in disentangling age-period-cohort (APC) effects [6]. Currently, national HEV control strategies remain fragmented, frequently failing to account for the complex interplay between demographic aging, zoonotic transmission dynamics, and genotype-specific pathogenicity [7].

HEV is predominantly transmitted via the fecal-oral route, with contaminated drinking water serving as the primary transmission vehicle in low- and middle-income countries (LMICs). In these resource-constrained regions, HEV epidemics are frequently precipitated by fecal contamination of drinking water sources, with outbreaks capable of affecting hundreds to thousands of individuals. In contrast, HEV infection is less endemic in regions with robust sanitation infrastructure and improved water quality; in such settings, cases are typically sporadic and linked to zoonotic transmission, most commonly associated with the consumption of undercooked meat from HEV-infected animals [8]. Emerging epidemiological evidence has also highlighted the potential role of alternative transmission routes, including blood transfusion and solid organ transplantation, which represent growing concerns particularly in high-income countries with aging populations. HEV remains a leading cause of acute sporadic viral hepatitis globally, with a disproportionate burden in developing countries characterized by inadequate sanitation and a high prevalence of chronic liver disease. The WHO has emphasized the critical importance of HEV prevention and control, with specific prioritization of high-risk groups such as the elderly [9].

Elderly individuals are increasingly recognized as a distinctively vulnerable population with heightened susceptibility to severe HEV infection and adverse clinical outcomes. Immunosenescence—the progressive age-related decline in immune function—impairs the ability to mount robust humoral and cellular immune responses against HEV, thereby compromising viral clearance and increasing disease severity. This immunological vulnerability is further compounded by the high burden of comorbidities in this age group [10,11]. Chronic liver disease, including cirrhosis and non-alcoholic fatty liver disease, is of particular epidemiological relevance; in individuals with pre-existing liver dysfunction, the liver’s capacity to tolerate additional pathogenic stress is significantly diminished, rendering HEV infection a life-threatening complication. HEV infection in elderly patients with underlying liver disease can precipitate acute-on-chronic liver failure (ACLF), a syndrome associated with alarmingly high short-term mortality rates [12]. Beyond liver-specific comorbidities, elderly individuals frequently suffer from other systemic conditions, including diabetes mellitus and cardiovascular disease, which not only compromise overall health status but also interact synergistically to exacerbate HEV-related liver injury and worsen clinical prognoses [13]. Collectively, the confluence of age-related immunosenescence and multiple comorbidities renders older adults prone to severe HEV infection and poor outcomes, establishing this cohort as a key priority for targeted HEV prevention and control efforts. International policy coordination and cross-border collaboration are imperative to address this global health challenge, particularly in resource-limited settings where the HEV disease burden is most pronounced.

Notably, public health research focus and resource allocation have historically been concentrated on younger populations, including women of childbearing age (WCBA), who are traditionally recognized as a high-risk group for HEV-related complications [14,15,16]. Despite this substantial attention to HEV infection in WCBA, HEV disease in elderly populations has remained relatively understudied and neglected [17,18]. While this emphasis on WCBA is merited, it has diverted critical attention from the growing burden of AHE in older adults. With the intensification of global aging, the public health significance of HEV infection in the elderly is becoming increasingly prominent. As noted, elderly individuals, due to compromised immunity and a higher prevalence of chronic diseases, are not only more susceptible to HEV infection but also at increased risk of severe disease, superinfection, and poor clinical outcomes [19]. Furthermore, the diagnosis of HEV infection in elderly patients is often challenging, as clinical manifestations may be non-specific and mimic those of other chronic conditions, leading to underdiagnosis and misdiagnosis. The unique epidemiological and clinical challenges posed by HEV infection in the elderly, coupled with their inherent physiological vulnerabilities and the dynamic nature of HEV transmission patterns, necessitate a critical reevaluation of current epidemiological methodologies and the development of age-specific intervention strategies. Consequently, there is an urgent and unmet need to strengthen targeted HEV prevention, detection, and control measures for elderly populations.

To address these critical research gaps, the present study leverages data from the Global Burden of Disease (GBD) Study 2021. Specifically, we delineated temporal trends in AHE among adults aged ≥ 65 years using case counts and age-standardized rates (ASR). Frontier analysis was employed to quantify the disparity between the observed HEV disease burden and the theoretical minimum burden achievable through optimization of the Socio-demographic Index (SDI). Age-period-cohort (APC) modeling was utilized to disentangle the independent effects of age, period, and cohort factors across 204 countries and territories worldwide. Additionally, multi-model forecasting—incorporating Bayesian APC (BAPC), NORDPRED, and Autoregressive Integrated Moving Average (ARIMA) models—was employed to project HEV disease trajectories up to 2050. The primary objectives of this study are to: (1) elucidate the epidemiological paradox of increasing AHE incidence concurrent with declining mortality rates among elderly populations; (2) identify high-risk subpopulations within the elderly cohort; and (3) develop targeted, evidence-based prevention and control strategies aligned with the United Nations’ Sustainable Development Goals (SDGs) for 2030.

## 2. Methods

### 2.1. Data Sources and Disease Definition

The Global Burden of Disease Study 2021 (GBD 2021), developed by the Institute for Health Metrics and Evaluation (IHME), provides a comprehensive and up-to-date epidemiological analysis of the disease burden associated with 371 conditions across 204 countries and territories. The GBD 2021 data used in this study are publicly accessible via the Global Health Data Exchange (GHDx) platform (https://ghdx.healthdata.org/, accessed on 20 December 2024). In accordance with the data sharing policy of the Institute for Health Metrics and Evaluation (IHME), non-commercial academic research is permitted to use, share, modify, or build upon these data without the need for additional permission [20]. This complies with the IHME FREE-OF-CHARGE NON-COMMERCIAL USER AGREEMENT, and detailed terms can be found on the IHME Terms and Conditions webpage. Diseases are categorized into a four-level hierarchy, with acute hepatitis E (AHE) classified at the fourth level under communicable diseases. In line with the International Classification of Diseases (ICD-10), hepatitis E virus infection is coded as B17.2. Within the GBD 2021 framework, AHE is defined as an infection caused by the hepatitis E virus, characterized by anti-HEV IgG seroconversion, regardless of symptom presence [12].

For this study, data on AHE among individuals aged 65 years and older were extracted from the GBD 2021 database. This included location-specific, age-specific, and sex-specific estimates for incidence, mortality, and disability-adjusted life years (DALYs), along with 95% uncertainty intervals (UIs). The analysis covered 204 countries with similar geographical and epidemiological contexts, stratified into seven age groups (65–69, 70–74, 75–79, 80–84, 85–89, 90–94, and ≥95 years) for both males and females.

The Sociodemographic Index (SDI) is a composite metric that evaluates the social development level of a region or country. It incorporates key factors such as economic income, educational attainment, and healthcare accessibility. The SDI is divided into five categories: low, low-middle, middle, high-middle, and high. This index helps policymakers and organizations identify areas requiring intervention and allocate resources effectively for sustainable development [21].

To estimate incidence, mortality, and infection rates, data from population-based studies and surveys on anti-HEV seroprevalence were utilized. Biases in non-reference data were addressed using Meta-Regression with Bayesian priors, Regularization, and Trimming (MRBRT) via logit-transformation. The epidemiological dynamics of AHE were modeled using a Bayesian framework tool, DisMod-MR 2.1 [22]. The incidence of anti-HEV IgG seroconversion derived from DisMod-MR was interpreted as indicative of AHE infection incidence.

### 2.2. Age Standardised Rate

To control for the effects of differing age distributions in the population, age-standardization was performed using the direct method [23,24]. We utilized the GBD global standard population distribution as the reference population. The age-standardized rates were computed for each condition of interest, ensuring comparability across populations and over time. $ASR=10^{5}\times \sum_{i=1}^{n}\left(\frac{C_{i}}{P_{i}}\times w_{i}\right)$, $C_{i}$: Number of cases in age group i, $P_{i}$: Total population of age group i, $w_{i}$: Weight of age group in the standard population, n: Total number of age groups.

### 2.3. Frontier Analysis

To evaluate the relationship between the burden of Hepatitis E Virus (HEV) in individuals aged 65 years and older and sociodemographic development levels, we utilized frontier analysis to develop a model based on age-standardized incidence rates (ASIR), mortality rates (ASMR), and disability-adjusted life years (DALYs) rates (ASDR), using the Socio-Demographic Index (SDI) as a key indicator. Unlike conventional regression approaches that primarily describe linear associations or predict outcomes, frontier analysis was chosen to address the complex, non-linear relationship between SDI and disease burden. This method also accounts for the multifaceted factors driving HEV-related morbidity and mortality in elderly populations.

Frontier analysis identifies the optimal achievable ASIR, ASMR, and ASDR values for each country or territory, given its current level of development. These theoretical minima serve as benchmarks for evaluating performance and setting realistic targets for disease burden reduction [25,26]. By comparing observed disease burden metrics with these theoretical benchmarks, the method quantifies the discrepancy between current outcomes and potential improvements. This approach not only highlights gaps in performance but also provides actionable insights into specific areas where interventions could yield the greatest impact on reducing HEV-related burden in elderly populations.

### 2.4. Age-Period-Cohort (APC) Model Analysis

The APC model is widely acknowledged as a sophisticated analytical framework that offers advantages over conventional methods in studies of health and socio-economic development [27]. This model enables the estimation of net drift and local drift, which reflect general time trends and specific time trends, respectively. Additionally, it quantifies the effects of three fundamental temporal dimensions: age, period, and birth cohort.

In applying the APC model, the age interval is typically aligned with the period interval, often using 5-year age groups matched with 5-year periods. For this study, data from the 2021 Global Burden of Disease (GBD) database were utilized, encompassing incidence rates of acute hepatitis E (AHE) among adults aged 65 years and older from 1990 to 2021, along with corresponding population data for each location. The adult population aged 65 years and older was stratified into seven age groups: 65–69, 70–74, 75–79, 80–84, 85–89, 90–94, and ≥95 years. The study period (1992–2021) was divided into six consecutive 5-year intervals: 1992–1996, 1997–2001, 2002–2006, 2007–2011, 2012–2016, and 2017–2021. Birth cohorts ranged from 1897–1901 to 1952–1956. The key components of the age-period-cohort (APC) model are as follows: 1. Net Drift: Represents the overall temporal trend, with statistical significance defined by a *p*-value ≤ 0.05. 2. Longitudinal Age Curve: Assesses changes in disease burden that are attributable to age-related effects. 3. Period Rate Ratios (RRs) and Cohort Rate Ratios (RRs): These metrics quantify period effects and birth cohort effects, respectively. An RR > 1 indicates a higher relative risk compared with the reference group, whereas an RR < 1 indicates a lower relative risk.

The overall time trend is quantified as the annual percentage change in incidence, referred to as net drift (% per year). This metric reflects the combined influence of calendar time and continuous birth cohorts. In contrast, the age-specific incidence trend, or local drift (% per year), captures the percentage change in annual incidence across different age groups. The APC model accounts for even minor shifts in drift values, which can significantly impact fitting rates over a 30-year period. The significance of annual percentage change trends was assessed using the Wald χ^2^ test, ensuring robust statistical evaluation [28,29]. Overall, this approach enables a nuanced understanding of long-term disease dynamics, facilitating informed public health interventions based on comprehensive trend analysis.

### 2.5. Prediction

The BAPC (Bayesian Age-Period-Cohort) model, NORDPRED model, and arima (AutoRegressive Integrated Moving Average) models were applied to forecast the incidence, mortality rate, and DALYs of acute hepatitis E in the elderly from 2022 to 2050.

The BAPC model is a Bayesian hierarchical model that decomposes health outcomes into age, period, and cohort effects. It estimates the contribution of each factor to disease burden while accounting for their interdependencies [30,31]. The NORDPRED model is a hierarchical Poisson regression framework designed for predicting disease burden in Norway. It incorporates spatial and temporal dependencies to improve prediction accuracy [32]. The ARIMA model is a time series forecasting method capturing linear dependencies [33,34]. It combines autoregressive, differencing, and moving average components to predict future trends.

### 2.6. Statistics

The age-standardized rate (ASR) was reported as the estimated value per 100,000 individuals, accompanied by its 95% confidence interval (CI). All statistical analyses and graphical visualizations were performed using R Studio software (version 4.2.1), with specific R packages 4.43 employed for key analytical steps: DisMod-MR for epidemiological dynamic modeling; APCtools for age-period-cohort (APC) analysis; rstanarm for constructing the Bayesian Age-Period-Cohort (BAPC) model, which adopted weakly informative priors (intercept term: N(0, 10); coefficients for age, period, and cohort effects: N(0, 5)), 4000 Markov Chain Monte Carlo (MCMC) iterations (2000 burn-in period, 2000 effective iterations) with convergence assessed by $\hat{R}$ < 1.05; nordpred for implementing the NORDPRED model; forecast for fitting the Autoregressive Integrated Moving Average (ARIMA) model, where annual data (1990–2021) were used, the optimal order (p,d,q) was determined via autocorrelation function (ACF), partial autocorrelation function (PACF), and Akaike Information Criterion (AIC), and the differencing order (d = 1) was selected based on stationarity tests; ggplot2 and sf for graph generation and geospatial data visualization; and metafor for auxiliary meta-regression analysis. All tests were two-tailed, with statistical significance defined as a *p*-value < 0.05.

## 3. Result

### 3.1. Global Trends in Acute Viral Hepatitis

The incidence absolute numbers and ASIR are shown in Table 1. The ASIR of acute viral hepatitis in the elderly population showed an overall downward trend from 1008.215 in 1990 to 890.605 in 2021, with a percentage change of −0.117 (−0.046 to −0.193). However, acute hepatitis C and acute hepatitis E depicted a contrasting trend. Especially for acute hepatitis E, the ASIR increased from 115.239 in 1990 to 116.981 in 2021, with a percentage change of 0.015 (0.0006 to 0.0296), showing the most significant increase.

**Figure 1:**
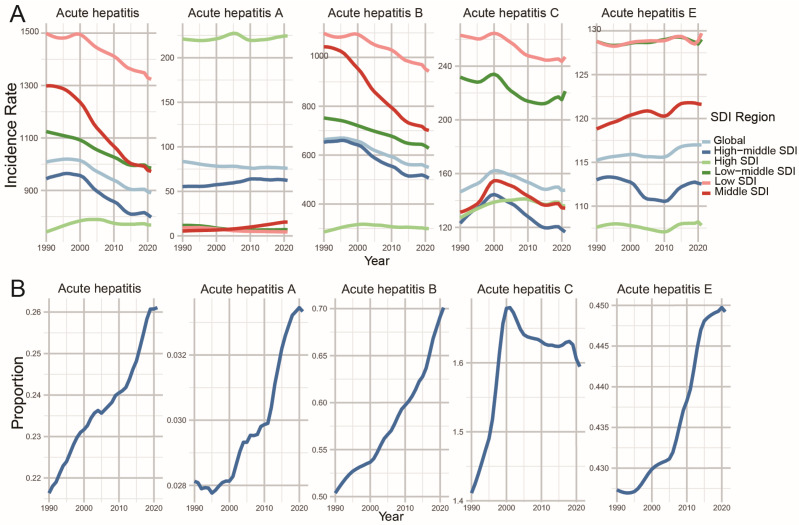
Age-standardized incidence rate of acute hepatitis E in global and the five SDI regions among individuals aged 65 and older (**A**) and the proportion of age-standardized incidence rate in individuals aged 65 and older relative to the total population (**B**) from 1990 to 2021.

Over the past three decades (1990–2021), the age-standardized incidence rate (ASIR) of the disease exhibited distinct variations across the five SDI quintiles. Except for high SDI regions, the overall incidence of acute viral hepatitis has shown a downward trend, with the most significant decrease in middle SDI regions. Notably, the ASIR of acute hepatitis E exhibited substantial temporal fluctuations over the study period. The middle SDI region showed the most significant increase compared to other categories of SDI, whereas the high-middle SDI regions have exhibited a volatile course, initially marked by a decline followed by a subsequent increase (Table 1 and Figure 1A).

Additionally, the proportion of acute viral hepatitis ASIR in individuals aged ≥ 65 years to overall individuals demonstrated a significant upward trend from 1990 to 2021. Specifically, this pattern was observed for acute hepatitis A, B, and E, with their respective proportions also showing a consistent upward trend during this period. For acute hepatitis C, the trend exhibited a distinct pattern: the ratio increased initially but subsequently decreased over time (Figure 1B).

### 3.2. Burdens by SDI Trend in Acute Hepatitis E

The global NetDrift for acute hepatitis E incidence was 0.044 (0.035 to 0.054) from 1990 to 2021. By SDI quintile, the APCs were 0.017 (−0.038 to 0.072) in low SDI areas, 0.023 (−0.003 to 0.05) in low-middle SDI areas, 0.064 (0.042 to 0.085) in middle SDI areas, −0.021 (−0.041 to 0.000) in high-middle SDI areas, and 0.007 (−0.007 to 0.021) in high SDI areas.

The mortality rate and DALYs of acute hepatitis E have declined significantly. The NetDrift for hepatitis E mortality was −3.114 (−3.421 to −2.805) globally, −3.465 (−4.151 to −2.774) in low SDI areas, −2.654 (−3.12 to −2.187) in low-middle SDI areas, −3.872 (−4.52 to −3.219) in middle SDI areas, −4.667 (−5.833 to −3.486) in high-middle SDI areas, and −1.301 (−2.985 to 0.411) in high SDI areas.

Similarly, the NetDrift for acute hepatitis E DALYs was −2.605 (−2.736 to −2.474) globally, −3.38 (−3.621 to −3.139) in low SDI areas, −2.487 (−2.697 to −2.275) in low-middle SDI areas, −3.197 (−3.426 to −2.969) in middle SDI areas, −3.392 (−3.677 to −3.106) in high-middle SDI areas, and −0.925 (−1.319 to −0.53) in high SDI areas (Table 2).

**Figure 2:**
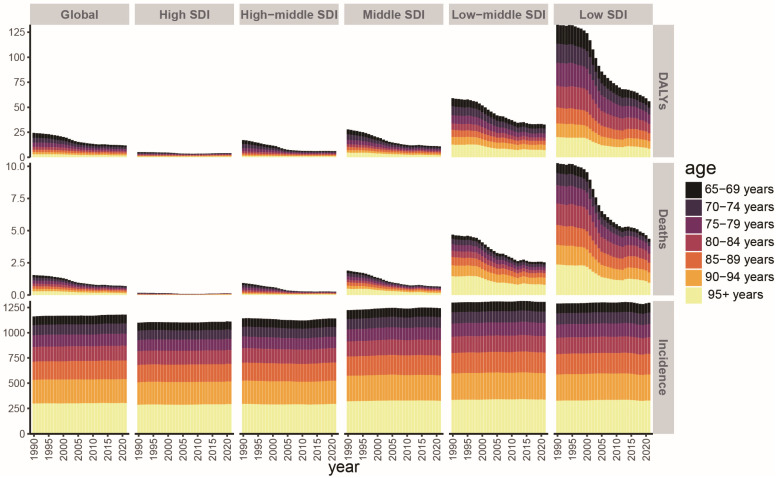
Incidence, mortality, and DALYs by age group in global and the five SDI regions from 1990 to 2021.

In terms of disease burden distribution across age groups, the 95+ years group bore the highest proportion of incidence, mortality, and DALYs, while the 65–69 and 70–74 age groups have a relatively lower burden (Figure 2).

### 3.3. Incidence, Mortality, and DALYs Trends Across Nations

**Figure 3:**
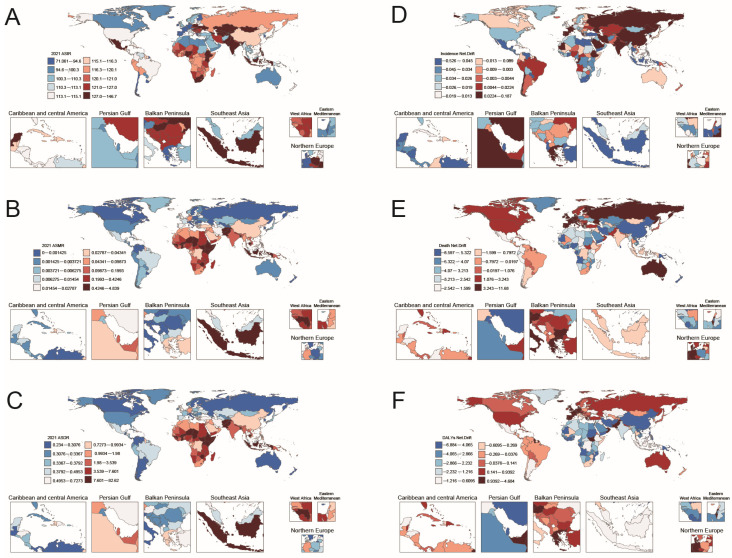
World map of (**A**–**C**) age-standardized incidence, mortality, and DALYs in 2021, and (**D**–**F**) the net drift of age-standardized incidence, mortality, and DALYs from 1990 to 2021.

In 2021, the incidence, mortality rate, and disability-adjusted life years (DALYs) of acute hepatitis E in the elderly in various countries were shown in Figure 3A–C and Supplementary Table S1. Countries exhibiting the highest incidence rates are predominantly distributed across Sub-Saharan Africa, Central Asia, and South Asia. Nations with the highest mortality rates and DALYs are chiefly located in Asia and Africa.

The APC model’s net drift shows diverse AHE incidence trends globally. Among 204 regions, 54 have rising incidence, with 4 having a net drift over 0.1%. Most of these regions are in high and high-middle SDI areas. China has the highest net drift at 0.187% yearly (0.137 to 0.236), with the 95% confidence interval lying entirely above zero. Greece follows at 1.383%, but the 95% confidence interval ranges from −0.02 to 0.284, crossing zero. India and Russia exhibit similar characteristics in incidence trends to China.

Regarding deaths, 31 regions see rising trends, with nine having a net drift exceeding 1%. Germany stands out with an 11.64% yearly rise (2.80 to 21.25), followed by Greece, Austria, and Algeria. For DALYs, 29 regions experience growth, eight with a net drift above 1%. Germany leads with a 4.41% annual increase (3.53 to 5.31), trailed by Greece, Austria, and Algeria.

Conversely, 147 regions witness declining incidence trends, 2 with a net drift below −0.1%. Italy is prominent with a −0.53% annual decrease (−0.63 to −0.42), followed by Albania, South Africa, and Yemen. For deaths, 147 regions face declining trends, 26 with a net drift below −5%. The Philippines leads with a −8.60% decrease (−13.30 to −3.64), followed by Iran, China, and Nigeria. In DALYs, 147 regions show downward trends, 11 with a net drift below −5%. Nigeria has the steepest drop of −6.42% (−7.66 to −5.17), followed by Bahrain, Yemen, and China (Figure 3D–F, Supplementary Table S2).

**Figure 4:**
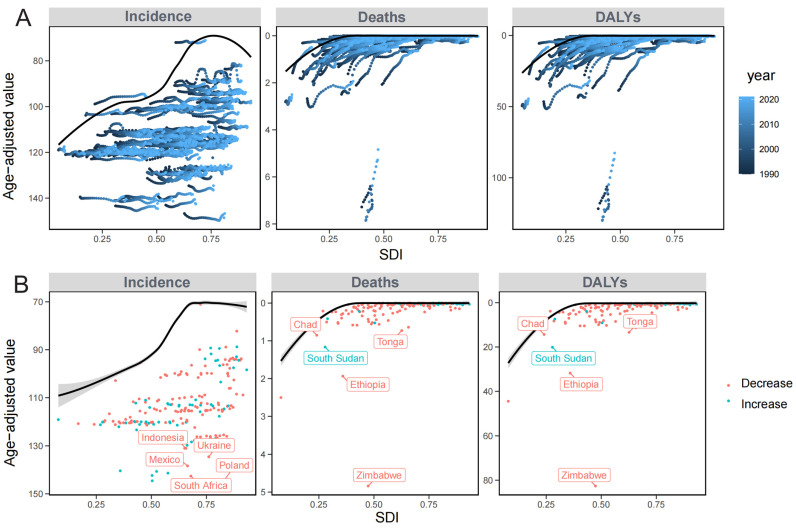
The Impact of Sociodemographic Transition on Incidence, Mortality, and DALYs of Acute Hepatitis E in the Elderly. (**A**) In acute hepatitis E among the elderly, the correlation between the Sociodemographic Index (SDI) and incidence, mortality, and Disability-Adjusted Life Years (DALYs). Consistent location markers indicate changes over 30 years. (**B**) The association between SDI and the Average Annual Percentage Change (EAPC) in incidence, mortality, and DALYs of acute hepatitis E among the elderly.

This study conducted a frontier analysis to assess the potential reduction of HEV burden, considering national and regional development levels. The results show a significant gap between incidence, mortality, and DALYs outcomes. Most countries have achieved frontier levels in ASMR and ASDR. However, the ASIR results are less positive, with many countries showing large gaps between current incidence rates and the frontier line. Notably, Zimbabwe also has considerable gaps between its mortality rates and DALYs and the frontier line (Figure 4A,B).

### 3.4. Age, Period and Birth Cohort Effects

**Figure 5:**
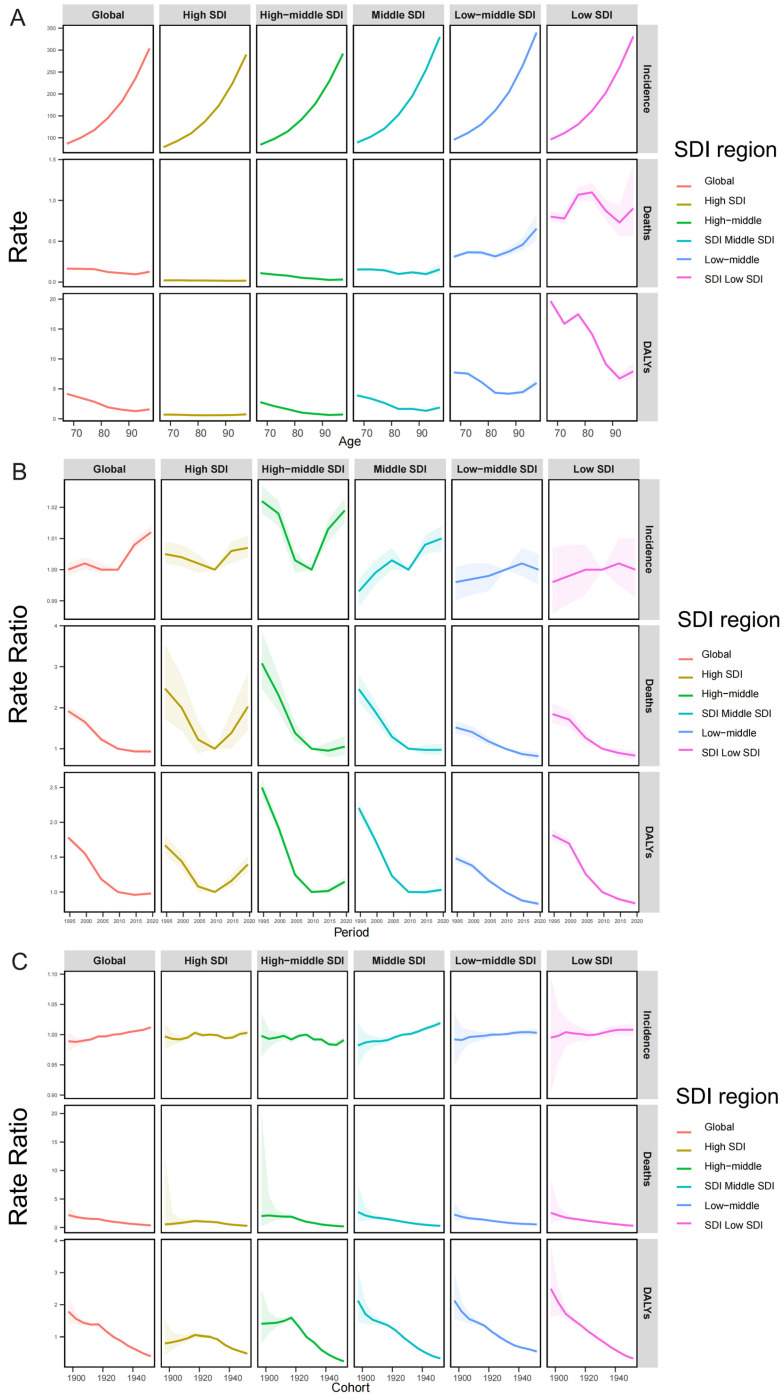
Impact of age, period, and birth cohort on AHE incidence, mortality, and DALYs in individuals aged 65 and older, analyzed using an APC model. (**A**) Age effects are described via age-specific longitudinal rates, adjusted for birth cohort differences and period-specific deviations. (**B**) Period effects are shown through the relative risk of AHE incidence across periods. (**C**) Birth cohort effects are demonstrated via cohort-specific relative incidence risks. Points and shaded areas indicate incidence rates or ratios and their corresponding 95% CIs. AHE = acute hepatitis E; SDI = sociodemographic index.

We analysed the age-period-cohort trends in incidence, mortality, and DALYs in AHE. For age-related impacts, different SDI regions show consistent patterns for incidence, with the lowest risk being observed in 65–70 years, followed by a growth in risk with increasing age. In low and low-middle SDI regions, the mortality risk and DALYs associated with AHE exhibit significant fluctuations across different age groups, unlike other SDI regions. Specifically, the mortality rate tends to increase sharply in low-middle SDI. In low SDI countries, mortality and DALYs remain high across all age groups (Figure 5A).

Globally, the period effect on the incidence rate demonstrates an overall upward trend despite declines in certain years, whereas mortality rates and DALYs have persisted in declining. In high and high-middle SDI regions, all exhibit an initial decline followed by a subsequent increase for incidence, mortality, and DALYs. Particularly, the mortality of high SDI regions reached the lowest in 2007–2011 period, followed by a significant increase (Figure 5B).

The birth cohort effect highlights the long-term influence of socio-demographic factors at birth on health. Globally, the incidence rate has exhibited a gradual upward trend, whereas the mortality rate and DALYs have demonstrated a progressive decline. The trends in most SDI regions are analogous to the global pattern. High-SDI exhibits a weak birth cohort effect, with minor differences in health indicators among cohorts. Meanwhile, low-SDI countries have seen marked improvements in mortality and DALYs, yet the incidence is rising (Figure 5C).

### 3.5. Prediction for Acute Hepatitis E to 2050

We applied the BAPC model to forecast the future trends of incidence rate, mortality rate, and DALYs of elderly AHE globally and by country. The incidence rate is projected to reach 113.04 (113.04 to 187.91) in 2050, showing an upward trend compared to 112.60 (112.47 to 112.83) in 2021 globally. Conversely, the mortality rate and DALYs are expected to decline, reaching 0.056 (−0.077 to 0.191) and 1.31 (−1.56 to 4.18), respectively, by 2050.

**Figure 6:**
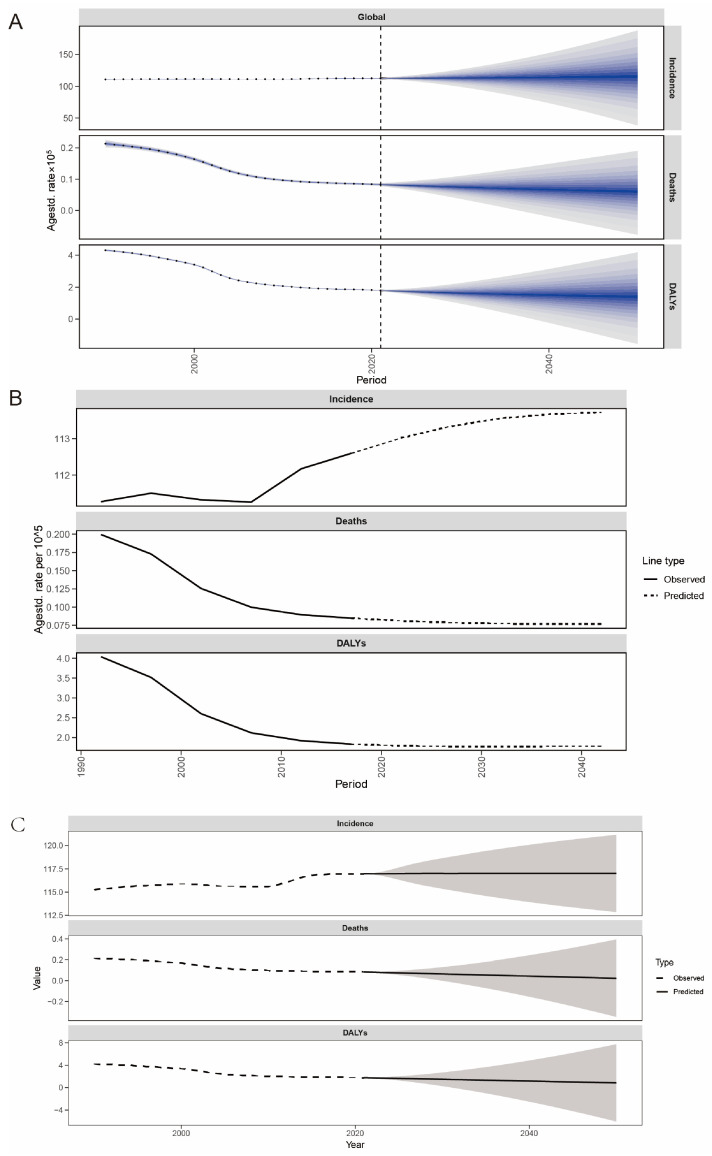
Projections of global acute hepatitis E age-standardized incidence rate, mortality rate, and DALYs among the elderly population from 2021 to 2050 using (**A**) BAPC, (**B**) nordpred, and (**C**) ARIMA models.

The global predictive trends of the NORDPRED and ARIMA models are analogous, characterized by a sustained escalation in incidence rates, whereas the mortality rates and DALYs demonstrate a declining trajectory. We further projected the 2050 global incidence, mortality rates, and DALYs for all countries using the ARIMA model (Figure 6A–C, Supplementary Table S3).

**Figure 7:**
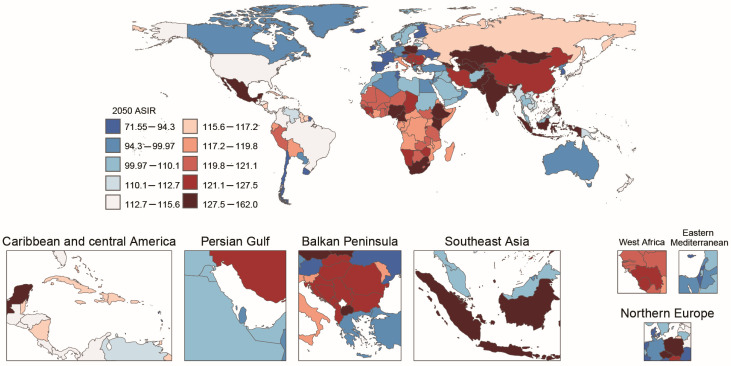
World map showing the projected global incidence rates of acute hepatitis E among elderly patients in 2050.

In 2050, high-incidence countries, similar to 2021, are predominantly located in Sub-Saharan Africa, Central Asia, and South Asia. China and India remain among the countries with high disease burden (Figure 7).

## 4. Discussion

This study reveals an upward trend in the incidence of acute hepatitis E (AHE) globally among the elderly. Data from the Global Burden of Disease (GBD) study shows a significant rise in age-standardized incidence rates (ASIR) of AHE in the elderly population from 1990 to 2021, contrasting with declining trends in other acute viral hepatitis types. Specifically, the ASIR of AHE increased from 115.239 in 1990 to 116.981 in 2021. We focused on AHE in the elderly due to its most significant incidence increase relative to the general population and other acute hepatitis types.

This increase in the elderly may stem from weakened immunity and a higher prevalence of chronic liver diseases [35,36], which render this population more susceptible to severe outcomes from HEV infection. Additionally, the global trend towards an aging population, especially in China, may exacerbate the disease burden in this vulnerable group [37,38,39]. The distinct variations in AHE ASIR across SDI quintiles reflect the influence of socio-demographic development on health outcomes, with middle SDI regions showing the most prominent increase, possibly due to rapid urbanization, changing dietary habits, and inadequate adaptation of public health measures.

Despite the rising incidence of AHE in the elderly, mortality rates and DALYs have decreased. For instance, populous nations like China and India exhibit high incidence rates but declining mortality and DALYs, likely due to advancements in medical technology, enhanced diagnostic capabilities, and improved public health infrastructure. Conversely, in some African countries such as Sudan and Egypt, mortality and DALYs remain elevated despite decreasing incidence rates, indicating deficiencies in disease management and resource allocation. Globally, the net drift for hepatitis E mortality was −3.114, and for DALYs, it was −2.605. The divergence between rising incidence and falling mortality and DALYs suggests that while new cases are increasing, disease severity and fatality are decreasing, possibly due to improved healthcare access and quality [40].

The 95+ years age group bears the highest disease burden, which highlights the need to focus on elderly populations, especially the oldest old, in hepatitis E management. This is attributed to cumulative immunosenescence and higher comorbidity rates in the oldest old, which increase their vulnerability to severe HEV infection and poor outcomes.

Geographically, high-incidence countries are concentrated in Sub-Saharan Africa, Central Asia, and South Asia, while high mortality and DALYs are mainly in Asia and Africa. China, India, and Russia have rising incidence trends, which is partly due to their large populations providing a vast “breeding ground” for disease transmission [36]. The high net drift in China (0.187%/year) may also be related to the rapid aging of its population and changes in zoonotic transmission pathways. In contrast, countries like Italy and South Africa show declining incidence, potentially due to effective sanitation improvements and targeted public health interventions.

Frontier analysis reveals that most countries have achieved theoretical minimum levels of mortality and DALYs but still have significant gaps in incidence prevention. This indicates insufficient efforts to prevent new HEV infections, especially in low and middle SDI regions with poor sanitation and low economic development. The lack of comprehensive vaccination programs, insufficient health education, and inadequate sanitation infrastructure in these regions contribute to the persistent high incidence rates [41,42]. Additionally, progress in medical technology has improved the diagnostic capabilities for hepatitis E, leading to the timely identification of more cases and, consequently, an increase in reported incidence rates [43]. Zimbabwe’s gaps in mortality and DALYs suggest the need for targeted interventions to improve clinical management alongside prevention efforts.

APC model analysis indicates significant effects of age, period, and cohort on AHE epidemiology. The lowest risk is observed in individuals aged 65–70 years, with risk increasing with age, which aligns with the physiological decline of the immune system and accumulation of comorbidities. Period effects show an overall upward trend in incidence rates, possibly influenced by global environmental changes, increased international travel, and changes in food production and consumption patterns. The initial decline followed by an increase in incidence, mortality, and DALYs in high and high-middle SDI regions requires vigilance, as it may indicate emerging transmission routes (e.g., blood transfusion, organ transplantation) or the emergence of new HEV genotypes.

Cohort effects reflect the long-term influence of socio-demographic factors at birth on health outcomes. High-SDI regions exhibit a weak birth cohort effect due to stable healthcare and socio-economic conditions, ensuring consistent protection across generations. Low-SDI countries have seen marked improvements in mortality and DALYs, likely due to economic development and improved healthcare access, yet the rising incidence still demands attention, highlighting the need to strengthen prevention-focused public health strategies.

Projections using the BAPC, NORDPRED, and ARIMA models consistently suggest that by 2050, the global incidence rate of AHE will continue to rise, while mortality rates and DALYs will decline. High-incidence regions such as Sub-Saharan Africa, Central Asia, and South Asia will still bear a heavy disease burden. China and India, as populous countries, will remain among high-burden nations despite declining mortality, which underscores the need for sustained investment in prevention and control. This projected rise in incidence urgently requires enhanced surveillance, improved sanitation, and expanded vaccination programs. The increasing trend in incidence could also be influenced by factors such as urbanization, climate change, and global travel, which may facilitate the spread of the virus in new regions.

This study has several limitations: database constraints precluded consideration of factors like HEV genotypes, environmental conditions, and seasonal variations [44,45]; discrepancies in data sourcing and quality may affect the accuracy of incidence and mortality rates in some countries; assumptions and parameters in predictive models, as well as limitations in historical data and the complexity of real-world scenarios, could influence the results and lead to uncertainties in projecting future trends (e.g., sudden changes in public health policies, emergence of new HEV strains, or unforeseen environmental changes may alter projected incidence rates and disease burden). Furthermore, as pointed out by the reviewers, the study lacks important information—HEV cases were identified by anti-HEV IgG seroconversion regardless of symptoms, which may introduce biases, including non-rare false positives in the aged population (potentially underestimating cases among subjects ≥ 65 years) and inadequate clinical information for seropositive subjects; additionally, pre-existing liver disease (e.g., autoimmune hepatitis) was not considered, even though HEV can cause acute-on-chronic liver failure in such patients, and serum hypergammaglobulinemia in autoimmune hepatitis patients may lead to false positive serological markers for HEV and other hepatotropic viruses, while the necessary exclusion of autoimmune hepatitis (per proposed diagnostic criteria) was not performed, further affecting result reliability [46,47].

## 5. Conclusions

The incidence of acute hepatitis E is rising globally, especially in the elderly, while mortality and DALYs are declining. Significant disparities exist across countries, and most nations need to enhance efforts to prevent new HEV infections. Future research should explore the impact of genotypes, environmental factors, and seasonal variations on HEV epidemiology and strengthen public health resource allocation and disease management strategies to mitigate the burden of AHE. Enhanced international cooperation, improved surveillance systems, and targeted vaccination campaigns are essential steps towards reducing the global burden of acute hepatitis E.

## Figures and Tables

**Table 1:** Global trends of acute hepatitis E incidence in adults 65 years and older from 1990 to 2021.

Cause	Incidence Number		Age-Standardized Rate per 100,000 Population		Percentage Change per 100,000 Population
	1990	2021	1990	2021	
Acute hepatitis	847,927.789 (572,283.071 to 1,258,119.934)	1,682,491.502 (1,130,618.567 to 2,423,854.641)	1,008.215 (674.967 to 1492.685)	890.605 (598.529 to 1283.122)	−0.117 (−0.046 to −0.193)
Acute hepatitis A	82,054.822 (61,976.651 to 104,028.044)	168,911.818 (123,405.878 to 220,148.749)	83.511 (59.898 to 111.943)	75.799 (53.533 to 101.577)	−0.093 (−0.048 to −0.137)
Acute hepatitis B	584,487.885 (304,641.667 to 975,192.117)	1,091,067.985 (562,688.380 to 1,847,235.916)	663.060 (337.805 to 279.053)	550.116 (279.053 to 942.793)	−0.1,703 (−0.170 to −0.339)
Acute hepatitis C	102,694.352 (59,865.037 to 165,227.660)	237,417.500 (138,914.191 to 375,155.142)	146.708 (85.837 to 235.455)	147.708 (87.073 to 233.453)	0.009 (0.0,015 to 0.017)
Acute hepatitis E	78,690.730 (42,526.190 to 133,613.723)	185,094.199 (99,026.678 to 314,087.772)	115.239 (62.190 to 193.536)	116.981 (62.692 to 196.410)	0.015 (0.0,006 to 0.0,296)

**Table 2:** Global and SDI trends of acute hepatitis E incidence, death and DALYs in adults 65 years and older from 1990 to 2021.

Location	1990		2021		1990–2021 APC
	Number (N, 95% UI)	Rate (per 100,000, 95% UI)	Number (N, 95% UI)	Rate (per 100,000, 95% UI)	NetDrift
	Incidence				
Global	78,690.73 (42,526.19 to 133,613.72)	115.24 (62.19 to 193.54)	185,094.20 (99,026.68 to 314,087.77)	116.98 (62.69 to 196.41)	0.044 (0.035 to 0.054)
low SDI	4564.99 (2329.27 to 7821.18)	128.81 (67.24 to 217.99)	10,285.77 (5352.84 to 17,487.32)	129.73 (68.37 to 218.44)	0.017 (−0.038 to 0.072)
Low-middle SDI	12,235.24 (6433.06 to 20,840.77)	128.79 (68.25 to 217.58)	31,067.82 (16,277.07 to 52,366.61)	129.02 (68.25 to 216.73)	0.023 (−0.003 to 0.05)
Middle SDI	19,618.56 (10,377.33 to 33,484.92)	118.81 (63.26 to 200.86)	58,365.02 (30,537.70 to 99,911.11)	121.61 (64.11 to 205.43)	0.064 (0.042 to 0.085)
High-middle SDI	19,629.66 (10,721.69 to 33,333.94)	113.00 (61.36 to 189.72)	42,366.86 (22,636.16 to 71,989.26)	112.53 (60.17 to 189.03)	−0.021 (−0.041 to 0.000)
High SDI	22,542.37 (12,130.11 to 38,250.12)	107.60 (57.25 to 180.79)	42,834.25 (22,943.99 to 72,393.54)	107.83 (57.29 to 180.73)	0.007 (−0.007 to 0.021)
	Death				
Global	165.74 (66.85 to 308.28)	0.21 (0.09 to 0.40)	141.28 (80.36 to 292.32)	0.08 (0.05 to 0.17)	−3.114 (−3.421 to −2.805)
low SDI	39.27 (11.77 to 101.41)	1.13 (0.35 to 2.94)	31.46 (12.78 to 71.12)	0.44 (0.17 to 1.01)	−3.465 (−4.151 to −2.774)
Low-middle SDI	43.06 (20.07 to 79.69)	0.45 (0.21 to 0.85)	56.02 (27.62 to 120.48)	0.23 (0.11 to 0.50)	−2.654 (−3.12 to −2.187)
Middle SDI	43.11 (16.74 to 83.53)	0.23 (0.09 to 0.44)	34.92 (18.79 to 73.76)	0.07 (0.04 to 0.15)	−3.872 (−4.52 to −3.219)
High-middle SDI	33.37 (8.08 to 75.97)	0.16 (0.04 to 0.37)	13.35 (7.01 to 23.93)	0.03 (0.02 to 0.06)	−4.667 (−5.833 to −3.486)
High SDI	6.87 (1.69 to 13.92)	0.03 (0.01 to 0.06)	5.46 (3.41 to 7.69)	0.01 (0.009 to 0.02)	−1.301 (−2.985 to 0.411)
	DALYs				
Global	3650.51 (1588.34 to 6589.33)	4.21 (1.87 to 7.60)	3340.84 (2021.51 to 6385.28)	1.78 (1.08 to 3.37)	−2.605 (−2.736 to −2.474)
low SDI	822.14 (249.06 to 2114.11)	19.70 (6.17 to 50.59)	654.41 (276.22 to 1448.66)	7.54 (3.11 to 16.84)	−3.38 (−3.621 to −3.139)
Low-middle SDI	922.26 (446.80 to 1670.35)	8.07 (3.90 to 14.86)	1233.87 (638.05 to 2565.19)	4.28 (2.22 to 8.95)	−2.487 (−2.697 to −2.275)
Middle SDI	957.34 (400.36 to 1802.14)	4.48 (1.90 to 8.39)	862.16 (524.14 to 1697.78)	1.52 (0.92 to 2.95)	−3.197 (−3.426 to −2.969)
High-middle SDI	748.91 (213.45 to 1638.71)	3.31 (0.99 to 7.26)	377.34 (233.44 to 626.71)	0.86 (0.54 to 1.40)	−3.392 (−3.677 to −3.106)
High SDI	198.33 (79.53 to 349.71)	0.79 (0.33 to 1.38)	211.07 (139.07 to 318.78)	0.49 (0.32 to 0.75)	−0.925 (−1.319 to −0.53)

## Data Availability

The original contributions presented in this study are included in the article/Supplementary Material. Further inquiries can be directed to the corresponding author.

## Supplementary Materials

The following supporting information can be downloaded at: https://www.mdpi.com/article/10.3390/tropicalmed11030082/s1. Supplementary Table S1. Incidence, mortality and DALYs of acute hepatitis E in the elderly in 2021 by country. Supplementary Table S2. Net drift of age-standardized incidence, mortality, and disability-adjusted life years (DALYs) across 204 countries, 1990–2021. Supplementary Table S3. ARIMA model forecasting the incidence, mortality and disability—adjusted life years of acute hepatitis E in the elderly in 2025.
